# Digital Economy and Health: Does Green Technology Matter in BRICS Economies?

**DOI:** 10.3389/fpubh.2021.827915

**Published:** 2022-01-14

**Authors:** Cuifeng Jiang, Hsuling Chang, Imran Shahzad

**Affiliations:** ^1^School of Economics, Qingdao University, Qingdao, China; ^2^Department of Accounting, Ling Tung University, Taichung City, Taiwan; ^3^UCP Business School, Faculty of Management Studies, University of Central Punjab, Lahore, Pakistan

**Keywords:** ARDL, digital economy, green technology, health, BRICS

## Abstract

The present study attempts to examine the impact of digitization and green technology on the health outcomes of BRICS countries over the period of 1993–2019. Internet users measure digitalization, and health outcome is determined by life expectancy. The study employed the ARDL estimation approach for empirical investigation of country-specific analysis. GDP per capita and current health expenditures have been incorporated as control variables. The study findings reveal that digitalization results in increasing life expectancy in the long-run in BRICS except for Brazil. While green technology tends to enhance life expectancy in the long-run in Russia and China, it produces an insignificant impact on health outcomes in the short-run. While GDP and health expenditures also improve life expectancy in mostly BRICS economies in the long-run and short-run. Our study provides some policy implications for BRICS nations.

## Introduction

Health sector significance and contribution in development are acknowledged at all phases of considerable decision making, particularly by the sustainable development goals (SDGs), as the third goal of SDGs is specified for good health and well-being. In view of the United Nations, the reason behind this goal is to “enable people to lead healthy lives and promote the well-being of all people at all ages” for the development of a flourishing society. The prosperous and flourishing society would then promote an efficient and effective health sector that reduces neonatal and infant mortality rates and increases the overall life expectancy (1).

Several agency reports and academic studies denoted that ICT development is capable to contribute to the healthcare and health sectors of developing countries (2). The use of the internet facilitates the delivery of health care services such as health-related services to nursing mothers. Furthermore, it also eases communication between health providers and patients (3). Internet use improves efficiency and management of public utilities (e.g., water delivery, mass transit) and government. ICT improves efforts regarding health literacy (4). For example, the internet delivers vast information to people. Furthermore, it permits people to obtain health-related information actively. ICT allows people to convey and exchange information without any temporal or spatial obstacles with low cost and high efficiency (5).

The significance of ICT in determining health outcomes is quite extensively explored in the literature. For example, radio technology-based projects in Africa attained enormous success in dropping the maternal mortality rate by providing health-related services to pregnant females (6). In view of Hoque et al. (7), after introducing mobile birth notification systems in Bangladesh, almost 89 percent of children's birth are taking place in public or private hospitals while previously almost 90 percent of childbirths were given recorded outside the hospital. Mobile phones increase the possibility of defending children and mothers from various diseases because of transferring information regarding various vaccination campaigns through sending direct messages. Mobile phones are also a common source of delivering important information about safety measures to prevent or control the extent of various illnesses. Dutta et al. (8) the study revealed that information technology significantly influences the chances of missing checkup appointments among pregnant females, significantly declining the chances of infant mortality rate.

According to Mechael (9), innovations and development in the healthcare system are immensely needed in developing economies. Furthermore, the progress in healthcare applications also required the development from other disciplines such as education, engineering, computer science, psychology, and sociology to enhance the satisfaction level of patients from the health care system (10). Several studies reported the potential of ICT in delivering health-related facts and its contribution to organizing several vaccination programs in developing economies (11). Experts also believe that ICT development can revolution the health sector of developing economies by improving the convenience of information. For instance, Rashid and Elder (12) encapsulated the healthcare situation of Uganda where the providers of health services saved almost 24 percent of the budget through the adoption of information technology in the process of data storage and collection.

Additionally, several studies exemplified that ICT contributes as a fundamental tool to attain the process of self-management by simplifying the way of communication among people and making the process more flexible for people to approach their doctors, relatives, and children (13). Due to development in the ICT sector, individuals get larger opportunities to communicate with each other for health-related services such as clinical decisions or provision of facilities for disease management. Moreover, ICT acts as a cost-effective instrument for promoting health-related services that enable individuals to share experiences and information among the population affected by mutual health issues (14).

Studies also denoted the significance of green technology in stimulating sustainable growth. Green technology means the identification of eco-friendly sources of growth, development of new eco-friendly industries, technological advancements, and jobs creation Guo et al. (15), which in return improves health. To obtain green growth, it is imperative to enhance innovations and investments that provide a base for sustainable growth and open new opportunities for human development. Hence, the development of the green economy needs extensive research on the circumstances of its development, system-related determinants, and its effect on the country's sustainable development (16). The procedures of green knowledge management contribute significantly to sustainable growth, most specifically in use, exchange, acquisition, and creation of knowledge, thus exerting significant influence on eco-innovations, green technologies, and socio-economic aspects of sustainable growth (17). Sustainable innovations enable companies to keep up with the pace of technological advancements. The study done by Das et al. (18) denoted that green technologies are aimed at producing such products that contribute to reducing environmental degradation by improving health.

The existing stock of literature quite extensively explored the impact of ICT and digital inclusion access on health outcomes at the global level. Moreover, the available stock of literature provides consensus-based clinical evidence and small sample-based country-specific evidence. Thus, there is a dire need to explore this nexus for longer period large samples. The above discussion also highlighted the importance of green technology in determining the health performance of the economy, but there is a lack of empirical evidence on this debate. In this perspective, the current study explores the impact of digitalization and green technology on health outcomes (19). It is highlighted that there is a lack of empirical studies that explored the impact of green technology and ICT development on health outcomes for BRICS economies (20). To fill this literature gap, this study investigates the impact of digital economy and green technology on the health outcomes of BRICS economies for a time period ranging from 1993 to 2019. To the best of the authors' knowledge, this is a pioneer study conducted to promote health outcomes in the case of BRICS economies. The findings obtained from this study will provide key essence to policymakers and practitioners in designing sustainable health-related policies.

## Model and Methods

Relying on the outcomes of previous theoretical and empirical studies (8, 21, 22) the following time series model is specified:


(1) $$\begin{array}{l} {\text{Health}}_{t\;}={\text{α}}_{0\;}+{\text{ α}}_{1}{\text{Internet}}_{t}+{\text{ α}}_{2}{\text{GT}}_{t}+{\text{ α}}_{3}GDP_{t}+{\text{ α}}_{4}HE_{t}\;\\ \;+{\text{μ}}_{t\;} \end{array}$$

Health is health outcomes, Internet is internet users, GT represents green technology, GDP represents the GDP per capita, and HE represents health expenditure. Previous literature studies [e.g., (8, 21)], have extended the health outcome model. ICTs has changed every aspect of human life, an estimate of α_1_ could be positive. Similarly, green technology is also reducing environmental pollution by increasing health efficiency in the long run, an estimate of α_2_ is expected to be positive. Since GDP and health expenditure are considered as key factors of health, respectively, estimates of α_3_ and α_4_ are expected to be positive. Equation (1) gives us only long-run estimates. We are also assessing the short-run effects of all concern variables; we must mold equation (1) into an error-correction model. The functional form is rewritten as the equation form as:


(2) $$\begin{array}{l} \begin{array}{c} \Delta {\text{Health}}_{t}=\gamma +{\sum^{\text{​}}}_{p=1}^{n1}\gamma_{1p\;}\Delta {\text{Health}}_{t-p}+{\sum^{\text{​}}}_{P=0}^{n2}\gamma_{2p\;}\\ \text{ Δ}{\text{Internet}}_{t-p}+{\sum^{\text{​}}}_{p=0}^{n3}\gamma_{3p\;}\Delta {\text{GT}}_{t-p}+{\sum^{\text{​}}}_{p=0}^{n4}\gamma_{4p\;}\\ \text{ Δ}{\text{GDP}}_{t-p}+{\sum^{\text{​}}}_{p=0}^{n5}\gamma_{5p\;}\Delta {\text{HE}}_{t-p}+{\text{π}}_{1}{\text{Health}}_{t-1}\\ \begin{array}{l} \;+\pi_{2}{\text{Internet}}_{t-1}+{\text{π}}_{3}{\text{GT}}_{t-1}+{\text{π}}_{4}{\text{GDP}}_{t-1}\\ \;\begin{array}{c} \;+{\text{π}}_{5}{\text{HE}}_{t-1}+\text{λ}.{\text{ECM}}_{t-1}+\;{\text{μ}}_{t} \end{array} \end{array} \end{array} \end{array}$$

Equation (2) is also an error-correction model, and long-run effects are reflected by π_1_− π_5_, but first differenced variables reported long-run effects error-correction model. Pesaran et al. (23) recommended two tests for cointegration. A standard F-test to test the null hypothesis: *H*_0_: π_1_ = π_2_ = π_3_ = π_4_ = π_5_ against the alternative of *H*_1_: π_1_≠0, π_2_≠0, π_3_≠0, π_4_≠0, π_5_≠0. If the calculated F-test statistic is significant, the alternative hypothesis is accepted, and the variables are said to be cointegrated. If a negative significant coefficient estimate is obtained from ECMt−1, it will support cointegration. The main advantage of the ARDL approach is that it allows us to blend I(1) and I(0) variables in the error-correction model. Furthermore, it also gives us short-run and long-run estimates in one step. This approach is suitable and better performs for a small sample size of data. The ARDL cointegration approach provides effective results in various orders of lags. Few additional diagnostic statistics are also applied to confirm the short and long-run estimates of ARDL (24). To test for serial correlation, we have used the Lagrange multiplier statistic as the LM test, while to test for heteroskedasticity, we report the Breusch-Pagan test statistic as the BP test (25). Model misspecification is tested through Ramsey's RESET statistic (26). To check the stability of long and short-run coefficient estimates, we can apply CUSUM and CUSUM-sq tests.

## Data

The study explores how the digital economy and green technology influence health outcomes in BRICS economies for a time span ranging from 1993 to 2019. Table 1 demonstrates the detailed information regarding symbols, definitions, and sources of variables. The dependent variable is health outcome which is measured by life expectancy at birth in total years. However, focused variables are the digital economy and green technology. The digital economy is measured by individuals using the internet in the percentage of the total population, while green technology is taken as the development of environment-related technologies in percent of all technologies. GDP per capita in constant 2015 US* and health expenditures as a percentage of GDP are used as control variables. Data for green technology is extracted from OECD, while data for remaining variables have been obtained from the World Bank. The detailed descriptive statistics of each variable for each country are reported in Table 2.

**Table 1:** Data definitions and sources.

**Variables**	**Symbol**	**Definitions**	**Sources**
Life expectancy	LE	Life expectancy at birth, total (years)	WDI
Internet users	Internet	Individuals using the Internet (% of the population)	WDI
Green technology	GT	Development of environment-related technologies, % all technologies	OECD
GDP per capita	GDP	GDP per capita (constant 2015 US*)	WDI
Health expenditure	HE	Current health expenditure (% of GDP)	WDI

**Table 2:** Descriptive statistics.

**Country**		**LE**	**Internet**	**GT**	**GDP**	**HE**
**Brazil**	Mean	72.07	28.82	9.402	8.940	8.282
	Std. Dev.	2.610	25.45	3.730	0.132	0.516
	Min	67.53	0.025	3.200	8.730	7.732
	Max	75.88	73.91	15.43	9.132	9.514
**Russia**	Mean	67.91	30.50	10.62	9.072	5.053
	Std. Dev.	2.786	31.11	1.760	0.286	0.238
	Min	64.46	0.013	7.310	8.614	4.743
	Max	73.08	82.64	15.71	9.403	5.638
**India**	Mean	64.88	7.064	8.362	6.931	3.789
	Std. Dev.	3.224	9.326	2.417	0.388	0.347
	Min	59.34	0.000	5.060	6.328	3.246
	Max	69.65	41.00	12.74	7.587	4.276
**China**	Mean	73.21	22.22	6.962	8.234	4.285
	Std. Dev.	2.358	22.68	1.377	0.669	0.508
	Min	69.49	0.000	3.730	7.122	3.572
	Max	76.91	64.56	9.440	9.233	5.351
**SA**	Mean	58.72	21.22	10.75	8.528	7.124
	Std. Dev.	3.708	22.98	2.673	0.121	0.714
	Min	53.44	0.114	6.330	8.340	6.098
	Max	64.13	68.20	15.80	8.658	8.253

## Results and Discussion

In this section, as a first step, we took assistance from lag selection criteria regarding the decision about the number of maximum lags to be applied in the regression process. To make a selection, we adopted the AIC method among all available methods. As we are dealing with annual data set, it is appropriate to choose three lags based on the AIC method. After selecting a number of lags, the next mandatory step is to check the stationarity properties of data. The findings of the ADF test confirm that our variables are either level stationary or first difference stationary. However, none of the variables is second difference stationary. Based on lag selection criteria and ADF unit root test findings, we can use the ARDL approach for further empirical investigation. Thus, Table 3 displays the short-run and long-run coefficient estimates of the ARDL approach.

**Table 3:** ARDL short and long-run estimates.

	**Brazil**	**Russia**	**India**	**China**	**South Africa**
**Short-run**					
D (Internet)	0.012^***^	0.026	0.011	0.010^**^	0.008^*^
	(2.736)	(1.327)	(0.943)	(2.377)	(1.911)
D [Internet (−1)]	0.001^**^				
	(2.395)				
D(GT)	0.003	0.039	0.002	0.003	0.001
	(0.708)	(0.555)	(0.247)	(0.172)	(0.115)
D(GDP)	0.042	2.788^**^	0.073	0.227^***^	0.914
	(1.028)	(2.294)	(0.708)	(4.880)	(0.940)
D [GDP (−1)]	0.108^***^		−0.115	0.098^**^	
	(2.590)		(1.104)	(1.961)	
D(HE)	0.007^*^	0.433	0.018	0.002	0.075^*^
	(1.737)	(0.884)	(1.067)	(0.515)	(1.737)
D [HE (−1)]	0.014^**^		0.123		
	(1.991)		(1.023)		
**Long-run**					
Internet	0.022	0.083^***^	0.416^***^	0.021^***^	0.008^**^
	(0.481)	(3.075)	(2.851)	(4.143)	(2.113)
GT	0.046	0.125^*^	0.051	0.011^*^	0.013
	(0.721)	(1.786)	(0.426)	(1.724)	(0.115)
GDP	2.063^***^	0.874	2.616^***^	2.516^***^	1.016^*^
	(3.210)	(0.252)	(8.943)	(4.016)	(1.803)
HE	2.598^**^	1.406	0.886	0.024^**^	1.884^*^
	(2.229)	(0.682)	(0.830)	(2.526)	(1.908)
C	7.836^**^	6.251^**^	3.732	5.473^***^	5.249
	(2.295)	(2.511)	(1.041)	(4.056)	(1.511)
**Diagnostics**					
F-test	12.32^***^	4.658^***^	15.25^***^	6.325^***^	8.356^***^
ECM (−1)	−0.372^**^	−0.308^*^	−0.290^**^	−0.364^***^	−0.410^***^
	(2.227)	(1.862)	(2.202)	(3.923)	(4.020)
LM	0.175	0.258	1.546	2.125	1.564
BP	0.452	0.689	0.546	1.457	1.023
RESET	1.542	1.023	1.035	0.879	1.356
CUSUM	S	S	S	S	S
CUSUM-sq	S	S	S	S	S

*** *p < 0.01*;

** *p < 0.05; and*

* *p < 0.1*.

Long-run coefficient estimates of the model reveal that the internet has a significant and positive impact on health outcomes in all BRICS economies except Brazil, confirming that the digital economy tends to increase life expectancy. For Lower Middle Income Countries, the investment and human capital development and skill upsurge the economic growth (27). Coefficient estimates reveal that in response to a 1 percent upsurge in internet users, life expectancy increases by 0.083 percent in Russia, 0.416 percent in India, 0.021 percent in China, and 0.008 percent in South Africa. Our finding infers that ICTs have more beneficial effects on individuals as well population heath. This also infers that ICT increases economic development, employment generation, and poverty alleviation, thus indirectly improving the population's health. ICT helps in e-health innovation and faster spreading of information through an effective and efficient transmission process, thus improving health. ICT is considered one of the cost-effective tools for health promotion, which permits individuals to share their experiences and information among the population. This finding is also consistent with Majeed and Khan (28), who infers that ICTs positively affect life expectancy. ICT also benefits health literacy efforts. This finding is also supported by Dutta et al. (8) and Alhassan and Adam (21). They infer that digitization can improve heath via different channels. Computer-aided diagnostics and treatment monitoring can be easily used through smart technology to improve health systems. A wide range of applications generally labeled “telemedicine” can be followed. The digital infrastructure can be used to inform the general population on healthcare.

Green technology tends to improve life expectancy in Russia and China, while in the case of other economies, green technology produces an insignificant impact on health outcomes. Findings reveal that a 1 percent increase in green technology increases life expectancy by 0.125 percent in Russia and 0.011 percent in China. This finding is also supported by Guo et al. (15), who confirm that green technology has lower pollution-related effects on ecosystems and health. We observed that green technology follows the environmental principle and law of green and digital economy. Green technology improves health by lowing energy consumption and eliminating environmental pollution in economic activities. Green technology offers a positive vision of sustainable development and health-promoting healthcare settings in the digital economy. Thus, our finding concludes that smart technology and green technology are important complements for health in a digital era.

In terms of control variables, GDP produces a significant and positive impact on life expectancy in all economies except Russia, while health expenditures result in increasing life expectancy in Brazil, China, and South Africa. ICT has a positive linkage with economic growth for Belt and Road economies (29). Similarly, technology innovation and adoption's marginal impact are muffled the Chinese pollution equation (30). Technology innovation initiates the economic growth and CO_2_ emissions for BRICS economies (31). Coefficient estimates show that 1 percent increase in GDP tends to increase life expectancy by 2.063 percent in Brazil, 2.616 percent in India, 2.516 percent in China, and 1.016 percent in South Africa, and 1 percent increase in health expenditures tend to increase life expectancy by 2.598 percent in Brazil, 0.024 percent in China, and 1.884 percent in South Africa.

Short-run findings of the model reveal that the digital economy exerts a significant and positive impact on life expectancy in Brazil, China, and Russia. Coefficient estimates reveal that in response to a 1 percent increase in internet utilization, life expectancy increases by 0.012 percent in Brazil, 0.010 percent in China, and 0.008 percent in South Africa. At the same time, green technology produces an insignificant impact on life expectancy in all BRICS economies. In the case of control variables, findings show that GDP results in increasing life expectancy in Russia and China, while health expenditures tend to increase life expectancy in Brazil and South Africa. In the lower panel of Table 3, coefficient estimates of some important diagnostic tests are given. These diagnostic tests are mandatory to confirm the validity of the findings of the ARDL model. Statistically significant coefficient estimates of F-statistics and ECM confirm that there exists a long-run cointegration association among variables of the model. A negative sign attached with the ECM value confirms that there is a tendency to converge toward equilibrium. Moreover, no autocorrelation and heteroskedasticity issue is found, as shown by the BP test and LM test findings. The findings of the Ramsey RESET test confirm that error terms are normally distributed. The stability condition is also fulfilled as confirmed by the findings of CUSUM and CUSUM-sq tests.

## Conclusion and Implications

The internet revolution has transformed the economies, and digitalization has done wonders for every economic sector. The dematerialization of the economy has only become possible due to digitalization. Dematerialization, on one side, has increased the process of economic growth. On the other side, it has also improved the environmental quality due to utilizing carbon-free energy resources. Just like other sectors, the internet revolution has also transformed the health sector. The internet has helped disseminate health-related information to a large number of people in a short period of time. People can now use the internet to collect health information and consult a doctor without any hassle. Therefore, we can predict the positive outcome of the internet on health outcomes. Consistent with this view, the present study attempts to examine the impact of digitization and green technology on health outcomes of BRICS countries over the period of 1993–2019. To get the short and long-run estimates of our variables, we have applied the ARDL model.

The estimates of the ARDL model confirm the positive impact of the internet on the life expectancy of Brazil, China, and South Africa in the short run and Russia, China, India, and South Africa in the long run. However, the long-run estimates of GT are significantly positive in the case of Russia and China only; whereas, the short-run estimates of GT are positive but insignificant in all countries. On the other side, the long-run estimates attached to GDP are positively significant in Brazil, India, China, and South Africa and the short-run estimates are positive and significant in the case of Brazil, Russia, and China. Lastly, the long-run estimated coefficients of HE is positive and significant in three of BRICS economies that include Brazil, China, and South Africa and the short-run estimates of HE are significantly positive in Brazil and South Africa. To ensure the validity of our results, we have also performed some diagnostic tests such as the LM test, Ramsey RESET, CSUSM, and CUSUM-sq. These tests confirm that our model is free from serial correlation, model misspecification, and parametric instability.

Technology itself is not a goal rather a tool to reach a goal. The foremost findings of the study are the positive association between digitalization and health outcomes in BRICS economies. These findings imply that the internet and green technology should be integrated with the existing structure and programs in the health sector to provide benefits to the end-users, and policymakers should focus on these lines. Moreover, the internet can become an important tool to bridge an information gap between providers of health services and those who utilize such services. Moreover, the interface of health-related websites and apps should be user-friendly so that maximum users can take benefit from such websites through the internet. Brazil should also follow the footprints of its BRICS allies to take benefit of the internet in the health sector by increasing the role of the internet in providing health services. However, green technology helps improve the health outcomes of only two BRICS economies, i.e., Russia and China. Green technology is quite helpful in improving the environment, but its role in the health sector is not fully revealed yet. The policymakers in BRICS countries should also focus on green technology while designing their health policies because green technology and the internet can significantly improve the environmental quality and health status in BRICS economies.

This research is limited to the analysis of digitalization, green technology, and health with respect to the BRICS. Future empirical research work should attempt to increase the scope and improve the consequences of health. Similarly, authors should analyze developed and developing economies. Future empirical studies can explore the role of smart technologies in the health industry. Moreover, future research can also offer a comparative-setting analysis for low and high-income economies. Such comparative-setting analysis empirical studies are more essential for targeted policy suggestions on health-related crises.

## Data Availability Statement

Publicly available datasets were analyzed in this study. This data can be found here: https://data.worldbank.org/.

## Author Contributions

CJ: conceptualization, software, data curation, and writing—original draft preparation. HC: methodology, writing—reviewing, and editing. IS: visualization and investigation. All authors contributed to the article and approved the submitted version.

## Conflict of Interest

The authors declare that the research was conducted in the absence of any commercial or financial relationships that could be construed as a potential conflict of interest.

## Publisher's Note

All claims expressed in this article are solely those of the authors and do not necessarily represent those of their affiliated organizations, or those of the publisher, the editors and the reviewers. Any product that may be evaluated in this article, or claim that may be made by its manufacturer, is not guaranteed or endorsed by the publisher.
